# Full-length transcriptome sequencing and methyl jasmonate-induced expression profile analysis of genes related to patchoulol biosynthesis and regulation in *Pogostemon cablin*

**DOI:** 10.1186/s12870-019-1884-x

**Published:** 2019-06-20

**Authors:** Xiuzhen Chen, Junren Li, Xiaobing Wang, Liting Zhong, Yun Tang, Xuanxuan Zhou, Yanting Liu, Ruoting Zhan, Hai Zheng, Weiwen Chen, Likai Chen

**Affiliations:** 1 Research Center of Chinese Herbal Resource Science and Engineering, Guangzhou University of Chinese Medicine; Key Laboratory of Chinese Medicinal Resource from Lingnan (Guangzhou University of Chinese Medicine), Ministry of Education; Joint Laboratory of National Engineering Research Center for the Pharmaceutics of Traditional Chinese Medicines, Guangzhou University of Chinese Medicine, Guangzhou, 510006 People’s Republic of China; 2 Guangdong Institute of Traditional Chinese Medicine, Guangzhou, 510520 People’s Republic of China

**Keywords:** *Pogostemon cablin*, Full-length transcriptome, Patchoulol, Expression profile, MeJA

## Abstract

**Background:**

*Pogostemon cablin* (Blanco) Benth. (Patchouli) is an important aromatic and medicinal plant and widely used in traditional Chinese medicine as well as in the perfume industry. Patchoulol is the primary bioactive component in *P. cablin*, its biosynthesis has attracted widespread interests. Previous studies have surveyed the putative genes involved in patchoulol biosynthesis using next-generation sequencing method; however, technical limitations generated by short-read sequencing restrict the yield of full-length genes. Additionally, little is known about the expression pattern of genes especially patchoulol biosynthesis related genes in response to methyl jasmonate (MeJA). Our understanding of patchoulol biosynthetic pathway still remained largely incomplete to date.

**Results:**

In this study, we analyzed the morphological character and volatile chemical compounds of *P. cablin cv.* ‘*Zhanxiang*’, and 39 volatile chemical components were detected in the patchouli leaf using GC-MS, most of which were sesquiterpenes. Furthermore, high-quality RNA isolated from leaves and stems of *P. cablin* were used to generate the first full-length transcriptome of *P. cablin* using PacBio isoform sequencing (Iso-Seq). In total, 9.7 Gb clean data and 82,335 full-length UniTransModels were captured. 102 transcripts were annotated as 16 encoding enzymes involved in patchouli alcohol biosynthesis. Accorded with the uptrend of patchoulol content, the vast majority of genes related to the patchoulol biosynthesis were up-regulated after MeJA treatment, indicating that MeJA led to an increasing synthesis of patchoulol through activating the expression level of genes involved in biosynthesis pathway of patchoulol. Moreover, expression pattern analysis also revealed that transcription factors participated in JA regulation of patchoulol biosynthesis were differentially expressed.

**Conclusions:**

The current study comprehensively reported the morphological specificity, volatile chemical compositions and transcriptome characterization of the Chinese-cultivated *P. cablin cv.* ‘*Zhanxiang*’, these results contribute to our better understanding of the physiological and molecular features of patchouli, especially the molecular mechanism of biosynthesis of patchoulol. Our full-length transcriptome data also provides a valuable genetic resource for further studies in patchouli.

**Electronic supplementary material:**

The online version of this article (10.1186/s12870-019-1884-x) contains supplementary material, which is available to authorized users.

## Background

*Pogostemon cablin* (Blanco) Benth. (Patchouli), a widely cultivated tropical and subtropical herb of Asia, is an important medicinal plant in Lamiaceae family due to its various clinical properties, including antimicrobial [1], antioxidant [2], analgesic, anti-inflammatory [3], antimutagenic [4] and antiemetic [5]. Patchouli plant have been extensively used in several traditional medicinal systems worldwide. In the traditional Chinese medicine (TCM) system, the dried aerial parts of patchouli known as ‘Guang-Huo-Xiang’ and Pogostemonis Herba, are frequently used to remove moisture, provide relief from summer heat and exterior syndrome, prevent vomiting and stimulate the appetite [6] which are documented in Chinese Pharmacopoeia. The raw herb is also widely used in numerous Chinese herbal compound prescriptions and Chinese patent medicine owing to its significant clinical efficacy. In addition, patchouli is also of great commercial importance due to its essential oil, which can be obtained through steam distillation of the shade dried leaves and is widely applied in fragrance industries to manufacture perfumes products, soaps and cosmetic products [7]. Therefore, due to the clinical and industrial need, there is a vastly increasing demand of *P. cablin*.

Modern phytochemical and pharmacological studies have indicated that patchouli oil is the major constituent obtained from the leaves of *P. cablin*. Of which, sesquiterpenes is the most abundant, mainly the patchouli alcohol (patchoulol, PA), a tricyclic sesquiterpene. Many studies have confirmed that patchoulol is the key constituent in regulating and controlling patchouli oil quality and largely contributes to the various biological activities of patchouli oil [8–11]. However, although with great medicinal value of patchoulol in patchouli, the functional genes related to patchoulol biosynthesis have not yet been fully identified. Recently, the high throughput, high accuracy and cost-effective NGS (Next-Generation RNA Sequencing) technology was widely utilized to identify and characterize genes in many non-model plants without the aid of a reference genome [12], and had been utilized for gene characterization at the transcriptome and genome level in *P. cablin* [13–16]*.* Using Illumina HiSeq 2000 platform, He et al. reported the leaf and stem transcriptome characteristic of *P. cablin* in which 108,996 unigenes were identified and many genes related to secondary metabolic production were characterized, and particularly 17 candidate genes were found involved in PA biosynthesis pathway [13]. Furthermore, the draft genome and complete chloroplast genome sequences of *P. cablin* were also revealed based on Illumina HiSeq 2500 platform, which would promote the phylogenic, population and genetic engineering research investigation of *P. cablin* [15, 16]. NGS has promoted the study of patchouli, however, the relatively short length of the reads generated from NGS restrict the yield of full-length genes [17], which brings problems in gene cloning and metabolic analysis. Moreover, in some cases, the low-quality transcripts generated by short-read RNA-seq sequencing can also lead to incorrect annotation. Thus, the global terpenoid biosynthesis pathway in *P. cablin* remains to be fully characterized.

Fortunately, with the development of sequence technology, the third-generation long-read sequencing has overcome these limitations, as it can construct full-length transcripts without an assembly step by providing amplification-free, single-molecule sequencing. SMRT (Single molecular real-time) sequencing performed with the PacBio Iso-Seq protocol is the most popular third-generation sequencing platform in most publications to date [18]. Because of its long-read sequencing feature, Iso-Seq has been widely used in full-length transcriptome analyses in numerous non-model organisms, including medical plants like *Astragalus membranaceus* [19], *Salvia miltiorrhiza* [20] and safflower [21],etc., and has become a powerful strategy for obtaining full-length gene sequences, reconstructing biosynthesis pathways, investigating alternative splicing, and validating and improving the existing genomes. In addition, combining NGS and SMRT sequencing can provide high-quality and more complete assemblies in genome and transcriptome studies [20, 22].

Herein, we combined morphological analysis, metabolic profile and transcriptome sequencing to acquire comprehensive information of the Chinese-cultivated *P. cablin cv.* ‘*Zhanxiang*’. To achieve a more complete view of *P. cablin* at transcriptional level, we generated full-length transcriptome of *P. cablin* from leaf and stem tissues via PacBio Iso-Seq. Functional annotations of the obtained full-length transcripts were systematically carried out, and the complexity of the biological processes and metabolic pathways in *P. cablin* were also revealed. Additionally, jasmonate (JA) and its derivative methyl jasmonate (MeJA) were reported to be general inducers of biosynthesis of plant secondary metabolite [23], but till now little is known about the expression pattern of genes especially patchoulol biosynthesis related genes in response to methyl jasmonate (MeJA). Therefore, with the full-length transcript models as a reference data, we established the transcriptome profiling of *P. cablin* induced by MeJA to analyze expression pattern of genes related to patchouli alcohol biosynthesis. The present work provided a better understanding of patchouli especially the molecular mechanism of patchoulol biosynthesis. Moreover, this transcriptome data also provide a valuable genetic resource for further study in patchouli.

## Results

### Morphology and volatile chemical components survey of the Chinese-cultivated *P. cablin*

*Pogostemon cablin* (Blanco) Benth. (Lamiaceae) is an herbaceous plant native to southern and southeast Asia [24], it is cultivated mainly in the Philippines, Indonesia, Malaysia, India, Brazil and China [25]. Here, we identified the Chinese *P. cablin* cultivar ‘Zhanxiang’, which is originally from Zhanjiang city in southern China and has been cultivated there for a long time (Additional file 1: Figure S1). With respect to morphology, the plant height of this species ranges from 87.05 cm to 109.30 cm, and length/width of leaf ranges from 1.73 to 1.88. Distinct stumpy and rhombic square stems were observed for *P. cablin cv.* ‘Zhanxiang’; compared with another Chinese *P. cablin* landrace [13] in Yangjiang city and the Indonesia germplasm, this cultivar also exhibits swelling at the nodes and a rather small ramification angle (Additional file 1: Figure S1).

Patchouli oil mainly consists of volatile components, which are the important and unique perfume bases in the fragrance industry and the active ingredients in drugs. To investigate volatile constituents in *P. cablin cv.* ‘Zhanxiang’, volatile chemical components in leaves were adequately examined by GC-MS with a variety of solvents, including chloroform (Additional file 2: Figure S2c), ethyl alcohol, ethyl acetate and n-Hexane (Table 1). In total, 39 of the characteristic components were identified, which may maximum number isolated so far, and most of them were sesquiterpenes. Important compounds including β-patchoulene, caryophyllene, α-guaiene, seychellene, β-guaiene, δ-guaiene, spathulenol and patchoulol were identified, in accordance with previous reports. We found that trans-caryophyllene, α-guaiene, seychellene, α-patchoulene, α-bulnesene, patchoulol, and squalene were found in all extracts. Among them, patchoulol (Additional file 2: Figure S2a) was the main sesquiterpene alcohol (C_15_H_26_O) medicinal constituent in *P. cablin.* We also detected another important medicinal constituent pogostone (4-hydroxy-6-methyl-3-(4-methylpentanoyl)-2H-pyran-2-one) in chloroform (Additional file 2: Figure S2b). These results provided informative insight into the medicinal utilization of patchouli and revealed a foundation for the biosynthesis of natural active constituent.

**Table 1:** Investigation of the volatile chemical constituents in *P. cablin* leaves using GC-MS

ID	Compound	CAS	Formula	Chloroform	Ethyl alcohol	Ethyl acetate	n-Hexane
1	1,5-Cyclodecadiene, 1,5-dimethyl-8-(1-methylethenyl)-, [S-(Z,E)]-	75,023–40-4	C_15_H_24_			○	
2	β-Elemene	515–13-9	C_15_H_24_				○
3	trans-Caryophyllene	87–44-5	C_15_H_24_	○	○	○	○
4	β-Caryophyllene	87–44-5	C_15_H_24_	○		○	
5	α-Guaiene	3691-12-1	C_15_H_24_	○	○	○	○
6	Seychellene	20,085–93-2	C_15_H_24_	○	○	○	○
7	1H-3a,7-Methanoazulene, 2,3,6,7,8,8a-hexahydro-1,4,9,9-tetramethyl-, (1.alpha.,3a.alpha.,7.alpha.,8a.beta.)-	560–32-7	C_15_H_24_	○	○	○	○
8	α-Gurjunene	489–40-7	C_15_H_24_	○	○	○	
9	α-Muurolene	31,983–22-9	C_15_H_24_			○	
10	Azulene, 1,2,3,3a,4,5,6,7-octahydro-1,4-dimethyl-7-(1-methylethenyl)-, [1R-(1.alpha.,3a.beta.,4.alpha.,7.beta.)]-	22,567–17-5	C_15_H_24_	○			○
11	Aciphyllene	87,745–31-1	C_15_H_24_	○		○	○
12	Azulene, 1,2,3,5,6,7,8,8a-octahydro-1,4-dimethyl-7-(1-methylethenyl)-, [1S-(1.alpha.,7.alpha.,8a.beta.)]-	3691-11-0	C_15_H_24_	○	○	○	○
13	Alloaromadendrene	25,246–27-9	C_15_H_24_		○		
14	(1R,4aS,6R,8aS)-8a,9,9-Trimethyl-1,2,4a,5,6,7,8,8a-octahydro-1,6-methanonaphthalen-1-ol	41,429–52-1	C_15_H_22_O	○		○	
15	(1S,1aS,1bR,4S,5S,5aS,6aR)-1a,1b,4,5a-Tetramethyldecahydro-1,5-methanocyclopropa [a]indene	52,617–34-2	C_15_H_24_			○	
16	(+) spathulenol	77,171–55-2	C_15_H_24_O	○		○	
17	Isoaromadendrene epoxide	1,000,159–36-6	C_15_H_24_O	○			
18	cis-Z-α-Bisabolene epoxide	1,000,131–71-2	C_15_H_24_O	○		○	
19	1,4-Dimethyl-7-(prop-1-en-2-yl)decahydroazulen-4-ol	21,698–41-9	C_15_H_26_O	○			
20	Patchouli alcohol	5986-55-0	C_15_H_26_O	○	○	○	○
21	4,7-Methanoazulene, 1,2,3,4,5,6,7,8-octahydro-1,4,9,9-tetramethyl-, [1S-(1.alpha.,4.alpha.,7.alpha.)]-	514–51-2	C_15_H_24_	○		○	○
22	4-Hydroxy-6-methyl-3-(4-methylpentanoyl)-2H-pyran-2-one	23,800–56-8	C_12_H_16_O_4_	○			
23	2H-Cyclopropa [g] benzofuran, 4,5,5a,6,6a,6b-hexahydro-4,4,6b-trimethyl-2-(1-methylethenyl)-	102,681–49-2	C_15_H_22_O	○			
24	(1R,4S,5S)-1,8-Dimethyl-4-(prop-1-en-2-yl)spiro[4.5]dec-7-ene	43,219–80-3	C_15_H_24_	○	○		
25	Caryophyllene oxide	1139-30-6	C_15_H_24_O			○	
26	1,4-Methanocycloocta [d] pyridazine, 1,4,4a,5,6,9,10,10a-octahydro-11,11-dimethyl-, (1.alpha.,4.alpha.,4a.alpha.,10a.alpha.)-	1,000,221–85-9	C_13_H_20_N_2_		○		
27	d-Nerolidol	142–50-7	C_15_H_26_O	○			
28	Farnesol	4602-84-0	C_15_H_26_O	○		○	
29	(+)-Aromadendrene	489–39-4	C_15_H_24_	○		○	
30	1,11-Hexadecadiyne	71,673–32-0	C_16_H_26_			○	
31	2,6,11,15-Tetramethyl-hexadeca-2,6,8,10,14-pentaene	38,259–79-9	C_20_H_32_	○		○	
32	1,2-Benzenedicarboxylic acid, bis(2-methylpropyl) ester	84–69-5	C_16_H_22_O_4_			○	
33	1,2-Benzenedicarboxylic acid, dibutyl ester	84–74-2	C_16_H_22_O_4_			○	○
34	2-(3,4-Dimethyloxyphenyl)-2,3-dihydro-5,7-dihydroxy-4H-1-benzopyran-4-one	1,000,395–79-6	C_17_H_16_O_6_	○			
35	Squalene	111–02-4	C_30_H_50_	○	○	○	○
36	Vitamin E	59–02-9	C_29_H_50_O2	○			
37	Oxiraneoctanoic acid, 3-octyl-, methylester	2500-59-6	C_19_H_36_O_3_		○		
38	γ-Sitosterol	83–47-6	C_29_H_50_O	○			
39	Bis(2-ethylhexyl) phthalate	117–81-7	C_24_H_38_O_4_			○	○

### Reconstruction of unique full-length transcript models of *P. cablin* via PacBio Iso-Seq

**Fig. 1:**
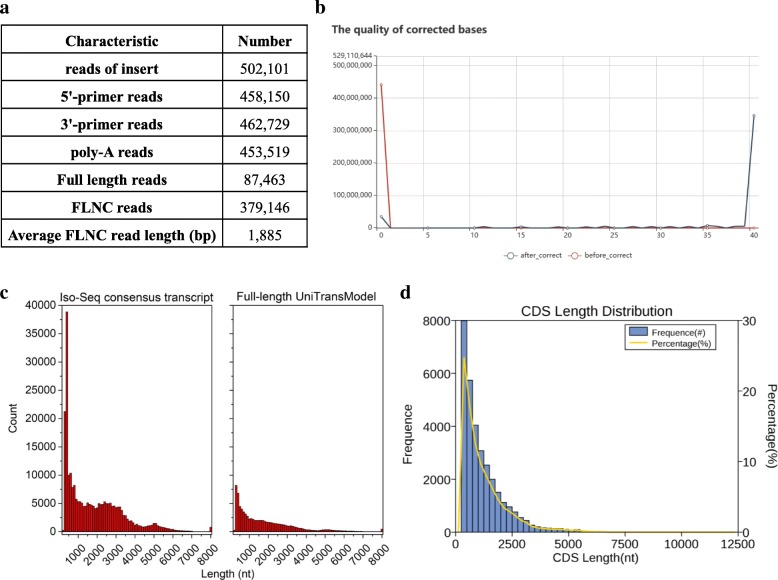
PacBio single-molecule long-read sequencing of *P. cablin*. **a** Summary of reads from PacBio Iso-Seq. **b** Comparison of quality from cluster consensus transcripts and corrected transcripts. **c** Length distribution of Iso-Seq consensus Transcripts and full-length UniTransModels. **d** Length distribution of CDS

Full-length cDNAs from pooled poly (A) RNA of two tissues (leaf and stem) were normalized and subjected for SMRT sequencing via the PacBio Sequel system to generate a full-length transcriptome of *P. cablin*. In total, 8,211,525 subreads (9.7 Gb) were generated from two SMRT cells, with an average read length of 1182 bp and N50 of 2205 bp. A total of 502,101 reads of insert (ROIs) were generated, of which, 387,463 sequences with two primers and poly-A tail were identified as full-length ROIs. The full-length ROIs were subsequently classified into chimeric and non-chimeric reads using the Iso-Seq pipeline, and 379,146 were identified as full-length non-chimeric (FLNC) reads with an average read length of 1885 bp (Fig. 1a).

By applying Iterative Clustering for Error Correction (ICE) algorithm and the final Arrow polishing algorithm to the above nonchimeric transcripts, we produced 251,015 full-length consensus transcripts for patchouli, including 26,155 polished high-quality (HQ, >99% accuracy) and 224,860 low-quality (LQ) transcripts. Additionally, the total length of these full-length consensus transcripts was 439,224,625 bp and was 3.6 times greater than that of the assembled unigenes via Illumina NGS in a previous study [13]. Compared with that of Illumina sequencing assembled unigenes, there were more sequences with >2000 bp length generated by Iso-Seq (Additional file 3: Table S1 and Fig. 1c). These results have demonstrated that PacBio Iso-Seq provides a pragmatic approach for producing full-length transcripts without the step of assembly, and it is an important improvement for transcriptome studies on species without a reference genome.

Performing error correction to acquire more accurate transcripts from Iso-Seq is necessary, since SMRT sequencing generates reads of several thousand bases but with a high error rate. While generating consensus transcripts via the pipeline of standard Iso-Seq bioinformatics, we applied self-correction by iterative clustering of circular-consensus reads. Afterwards, we used Proovread software to correct the errors of both HQ and LQ consensus transcripts, and the quality of the corrected bases is shown in Fig. 1b. Subsequently, CD-HIT-EST (c = 0.99) was used for further clustering to obtain the non-redundant transcripts [26], we successfully reconstructed a total of 82,335 full-length UniTransModels for pooled samples of patchouli leaves and stems. In this pipeline, we observed that the numbers of transcripts were significantly reduced, while the length distributions of the full-length UniTransModels were similar to the consensus transcripts.

### Functional annotation

Transcripts in *P. cablin* were functionally annotated and classified via BLASTx or tBLASTx according to sequence similarities (E-value ≤1e-5) against seven different protein and nucleotide databases. A total of 65,826 (79.95%) UniTransModels were successfully matched to known proteins in at least one out of the seven databases, and the successful rates in each single database ranged from 18.27 to 71.89% (Table 2). The homologous species of *P. cablin* were predicted by aligning sequences to the Nr database, and the largest number of sequences was distributed in *Sesamum indicum* (34,662), followed by *Erythranthe guttata* (9827) and *Dorcoceras hygrometricum* (1910) (Additional file 4: Figure S3).

**Table 2:** Summary of functional annotation results for *P.cablin*. UniTransModels to public databases

Annotated databases	Nr	SwissProt	KEGG	KOG	GO	Nt	Pfam	Total
Transcript Number	59,188	53,172	57,164	33,793	15,036	46,110	15,036	65,826
Percentage (%)	71.89	64.58	69.43	41.04	18.26	56	18.26	79.95

**Fig. 2:**
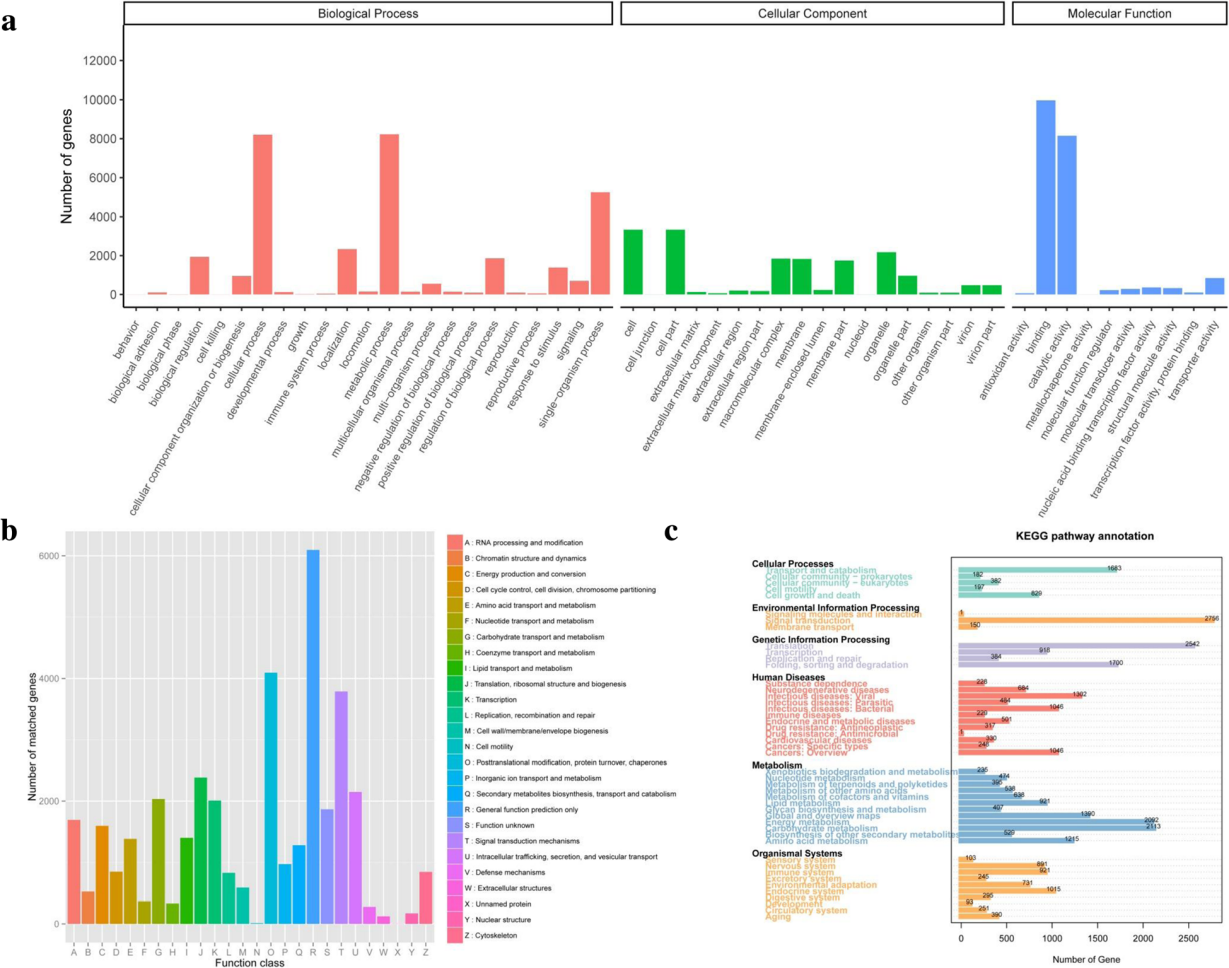
Functional annotation and classification of *P. cablin* UniTransModels. **a** GO classification of *P. cablin* UniTransModels. **b** KOG functional classification of *P. cablin* UniTransModels. **c** KEGG pathway  classification of *P. cablin* UniTransModels

In functional classification, GO terms were assigned to each UniTransModels via BLAST2GO program base on annotation of Nr database. Overall, there are 15,036 transcripts were assigned into the three major categories (biological process, molecular function and cellular component; Fig. 2a). Major representatives in classification of biological processes were “cellular process” (GO:0009987) and “metabolic process” (GO:0008152). The major subgroups of cellular component were “cell” (GO:0005623) and “cell part” (GO:0004464). For molecular function category, “binding” (GO:0005488) and “catalytic activity” (GO:0003824) were represented most.

A total of 33,793 UniTransModels were assigned to 24 functional clusters in KOG. The largest three categories were “general function prediction only” (18.04%, 6095), “posttranslational modification, protein turnover, chaperones” (12.12%, 4095), “signal transduction mechanisms” (11.21%, 3787). Secondary metabolites contribute a lot to the pharmacological activity and quality of Patchouli, and 1280 (3.79%) UniTransModels were clustered into the “Secondary metabolites biosynthesis, transport and catabolism” category (Fig. 2b).

For exploration of biological functions and interactions, Patchouli UniTransModels were queried against the KEGG. Totally, 57,164 transcripts were annotated and assigned to 363 KEGG pathway annotations (KOs) which involved six functional categories (Fig. 2c). Among these pathways, the “Metabolism” pathway included the most isoforms, and represented 24.9% of our PacBio data sets; in which “Carbohydrate metabolism” and “energy metabolism” were the most represented pathways observed (both representing 3.7%). “Metabolism of terpenoids and polyketides” was assigned to 395 of these transcripts. Together, the above results from the functional annotation and classification of the UniTransModels, not only reveal a comprehensive functional characterization for the full-length *P. cablin* transcriptome to us, but also provide a great help in further research on gene function.

### Coding sequence prediction and annotation

Protein-coding sequences (CDSs) were determined from the UniTransModels via the ANGEL pipeline. We identified a total of 32,232 CDSs, among which, 12,323 (38%) were longer than 900 nt (Fig. 1d). The CDSs were subsequently compared to the protein sequences of Viridiplantae, Arabidopsis and rice curated in UniProtKB to acquire a wide-ranging functional annotation of the *P. cablin* full-length transcriptome. Approximately 73.7% of the CDSs were annotated via aligning sequences from these well-curated databases (Table 3, Additional file 5: Table S2), and the remaining 1272 unannotated CDSs might represent novel *P. cablin* species-specific genes.

**Table 3:** Number of annotated CDS of *P. cablin.* in UniProtKB

	Number of CDS	Percentage (%)
UniPortKB_ *viridiplantae*	23,717	73.58
UniProtKB_*Arabidopsis_thaliana*	21,778	67.57
UniProtKB*_Oryza_sativa*	21,166	65.67
Total	23,754	73.7

### Identification of transcription factor and lncRNA

**Fig. 3:**
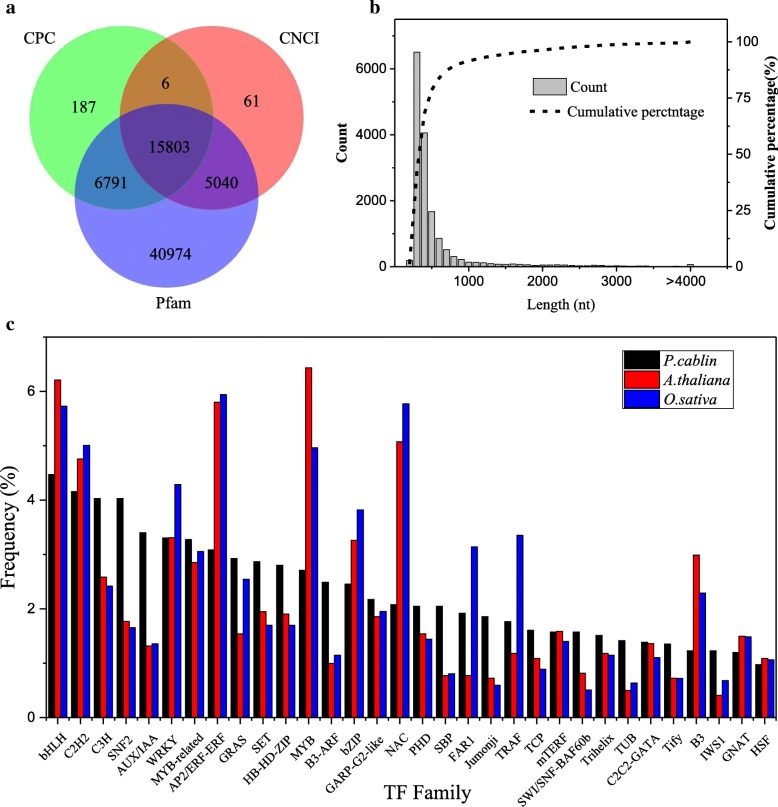
Identification of lncRNAs and Transcription Factors (TFs) in *P. cablin* transcriptome. **a** Venn diagram of the number lncRNAs predicted by CPC, CNCI and Pfam protein structure analysis. **b** Length distribution of lncRNAs of *P. cablin*. **c** Comparison of the primary transcription factor families identified in *P. cablin* transcriptome to which in *Arabidopsis thaliana* and *Oryza sativa*

Transcription factors (TFs) and Transcription regulators (TRs), vital elements in gene regulatory networks, are prevalent in all living organisms and many different kinds of biological processes including growth, development and environmental responses [27]. By applying our sequencing data to the software iTAK, we annotated 3176 TFs belonging to 90 different families totally. Compared with those in *A. thaliana* and *O. sativa*, isoforms of the C3H, MYB-related, GRAS, B3-ARF and SBP families were more abundant in *P. cablin*. TRs such as SNF2, SET, AUX/IAA and Jumonji families also had a particularly higher frequency of isoforms in *P. cablin* (Fig. 3c).

Aside from protein-coding RNAs, noncoding RNAs are a main consitiuent of the transcriptome. As key functional regulators in a wide spectrum of biological processes, lncRNAs have become an emerging important area of research in molecular biology. In this study, a total of 15,803 candidate lncRNAs were identified by three methods from 82,335 UniTransModels in *P. cablin* (Fig. 3a). Most (91.5%) of these lncRNAs were shorter than 1000 bp, and only 62 (0.4%) were longer than 4000 bp (Fig. 3b). Only seven of these lncRNAs have high homology with known pre-miRNAs by querying against the miRBase; among them, three lncRNAs served as precursors for miR159, two for miR166a and the rest two respectively for miR172 and miR2916 (Additional file 6: Table S3).

### Identification of candidate genes involved in patchoulol biosynthesis

*P. cablin* is rich in terpenoids, in this study, based on the KEGG pathway assignments, we found a total of 395 transcripts were assigned to the “metabolism of terpenoids and polyketides” in our full-length sequences; among them, 152 were assigned to the terpenoid backbone (ko00900), 12 to monoterpenoid (ko00902), 47 to sesquiterpenoid and triterpenoid (ko00909) and 34 to diterpenoid biosynthesis (ko00904) (Additional file 7: Table S4).

**Fig. 4:**
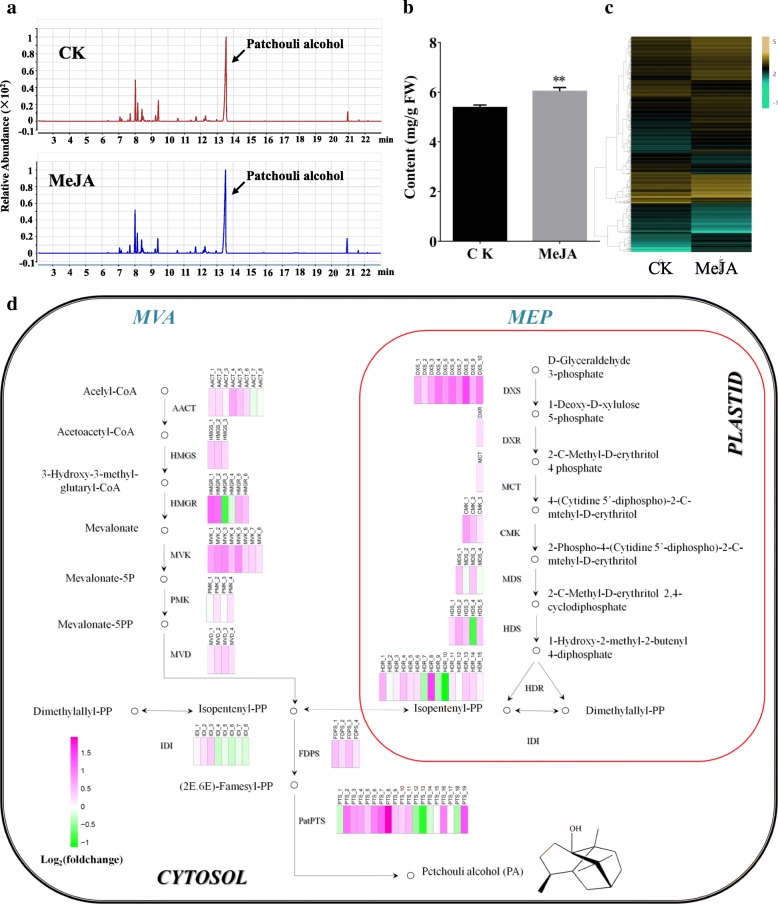
Patchoulol content and genes expression pattern in patchouli leaves induced by MeJA. **a** GC-MS chromatogram of leaves extracts from plant sprayed with or without MeJA. **b** Contents of patchoulol in leaves significantly increased after MeJA treatment. The amount of patchoulol was first calculated comparing with external standard and then dividing by the fresh weight of the samples. Error bars indicate the SD of three biological replicates. ** indicates significant difference of the means at *p* < 0.01 between non-treantment (CK) and MeJA-treatment leaves for each parameter mesureed (*n* = 3). **c** Abundance of DEGs in leaf tissues in response to MeJA treatment. The heat map showing genes having a fold change ratio ≥ 2, with corrected *p*-value<0.01. **d** Abundance changes of transcripts involved in the cytosolic MVA, plastidial MEP, and *P. cablin* patchoulol biosynthesis pathways in response to MeJA. The expressions are indicated by heat map, estimated using Log_2_(foldchange) value for each transcript. Magenta means high expression and green means low expression, the color gradually from green to magenta represents gene expression abundance from low to high

Patchoulol, the major components in patchouli oil, belongs to sesquiterpenes. Its biosynthesis includes terpenoid backbone biosynthesis and sesquiterpenoid biosynthesis pathways. All known that in plant, all terpenoids including patchouli alcohol are originated from the C5 unit isopentenyl diphosphate (IPP), which can be synthesized through the cytosolic mevalonate (MVA) pathway and plastidial methyl-erythritol phosphate (MEP) pathway. In *P. cablin* full-length transcriptome, 32 transcripts encoding six enzyme genes were identified to be involved in MVA pathway, including 8 AACT (acetyl-CoA acyltransferase, EC 2.3.1.9) genes, 3 HMGS (3-hydroxy-3-methylglutaryl-CoA synthase, EC 2.3.3.10) genes, 5 HMGR (3-hydroxy-3-methylglutaryl-CoA reductase, EC 1.1.1.34) genes, 8 MVK (mevalonate kinase, EC 2.7.1.36) genes, 4 PMK (phosphomevalonate kinase, EC 2.7.4.2) genes and 4 MVD (mevalonate 5-dinhophate decarboxylase, EC 4.1.1.33) genes. In addition, 39 transcripts encoding seven enzymes were also found in the MEP pathway, including 10 DXS (1-deoxy-D-xylulose 5-phosphate synthase, EC 2.2.1.7) genes, 1 DXR (1-deoxy-D-xylulose 5-phosphate reductoisomerase, EC 1.1.1.267) gene, 1 MCT (MEP cytidyltransferase, EC 2.7.7.60) gene, 3 CMK (4-(Cytidine 5-diphospho)-2-C-methylerythritol kinase, EC 2.7.1.148) genes, 4 MDS (2-C-Methy-D-erythritol 2,4-cyclodiphosphate synthase, EC 4.6.1.12) genes, 5 HDS (hydroxymethylbutenyl 4-diphosphatesynthase, EC 1.17.7.1) genes and 15 HDR (4-hydroxy-3-methylbut-2-enyldiphosphatereductase, EC 1.17.1.2) genes (Fig. 4d). In this work, with the exception of DXR and MCT, the remaining synthase-enzyme genes in the MVA and MEP pathways displayed multiple transcripts, presenting in the transcriptome as multiple paralogous genes. Additionally, we also found 8 transcripts encoding IDI (isopentenyl diphosphate isomerase, EC 5.3.3.2), which catalyzes the conversion of the common precursor IPP to DMAPP (Dimethylallyl diphosphate). Subsequently, with the catalysis of FDPS (farnesyl diphosphate synthase, EC 2.5.1.10), IPP and DMAPP are condensed to form farnesyl diphosphate (FPP), which is further catalyzed by patchoulol synthase (*PatPTS*) to produce patchouli alcohol. In this full-length transcriptome, 4 and 19 transcripts were identified to encode FDPS and *PatPTS*, respectively (Fig. 4d).

### Expression analysis of patchoulol biosynthesis genes under MeJA treatment

Jasmonic acid (JA) is a kind of plant hormone which involving in many physiological processes of plant during growth and development, including secondary metabolism [28]. In this study, the patchoulol content of *P. cablin* leaves significantly increased after MeJA treatment (Fig. 4a, b). To investigate the gene expression patterns, transcriptome profiling of *P. cablin* leaves under MeJA treatment were further performed with a reference transcript of our *P. cablin* full-length transcript models, and the relative gene expression level was normalized as FPKM. Results showed that a total of 655 DEGs were identified with at least a two-fold change in response to MeJA treatment (Fig. 4c and Additional file 8: Table S5), of which 427 were up-regulated and 228 were down-regulated, indicating that more activation than suppression of genes induced by MeJA.

We further detected the expression patterns of genes related to patchoulol biosynthesis in response to MeJA treatment. As shown in Fig. 4d and Additional file 9: Table S6, most of the genes in the MVA and MEP pathways were up-regulated after MeJA treatment, in accord with the increasing tendency of patchoulol content. Particularly, transcripts encoding HMGR (HMGR_1 and HMGR_2) in the MVA pathway and encoding DXS (DXS_4, DXS_6, DXS_8) and HDR (HDR_8) in the MEP pathway were clearly up-regulated with at least two-fold change under MeJA treatment, suggesting that these genes are key regulatory factors in the biosynthesis of patchoulol in *P. cablin*. IDI converts the IPP to DMAPP, and then IPP and DMAPP were condensed to generate FPP, the common precursors of sesquiterpenes, by FDPS. In our gene expression profile data, the relative expression of IDI multi-transcripts were varied, and all were insensitive to MeJA. In addition, four transcripts encoding FDPS were all up-regulated in response to MeJA, although the fold change was less than two; especially, FDPS_3 had a very high expression level, which thereby becomes noteworthy. Patchoulol synthase (*PatPTS*) was detected as a multi-isoform that was mainly up-regulated in response to MeJA, which was also consistent with the increasing of patchoulol content. Among them, PTS_2, PTS_7, PTS_8, PTS_19 were up-regulated with a fold-change more than two; notably, PTS_3, PTS_4, PTS_5, PTS_6, PTS_9 and PTS_11 were exhibited with a very high expression level. In brief, majority of genes related to patchoulol biosynthesis were up-regulated in response to MeJA treatment, which had a positive correlation with the patchoulol content. However, experiment evidence for gene functional specialization in production of patchoulol still need further study.

### Expression analysis of genes associated with the JA synthesis and signaling pathway

**Fig. 5:**
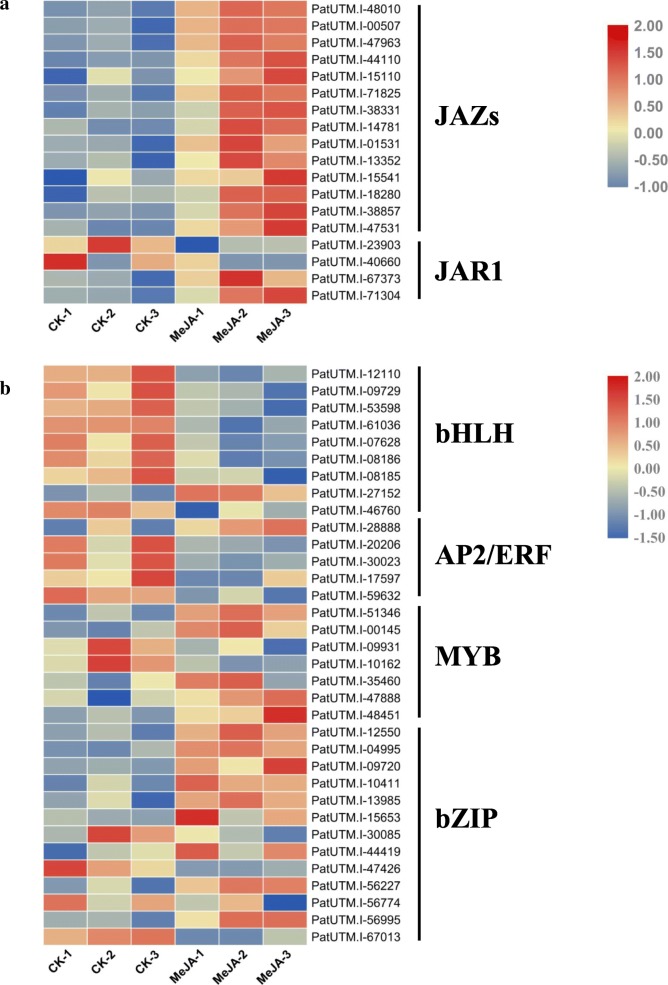
Expression profiles of genes associated with JA synthesis and signaling in patchouli induced by MeJA (foldchange > 1.5. *p*-value ≤0.05). **a** Heat map depicting abundance change of JAZ- and JAR1 co-orthologues. Blue color in the map represents low transcript abundance and red represents high level of transcript abundance. The same below. **b** Heat map depicting differential expression pattern of transcription factors in *P. cablin* induced by MeJA

**Fig. 6:**
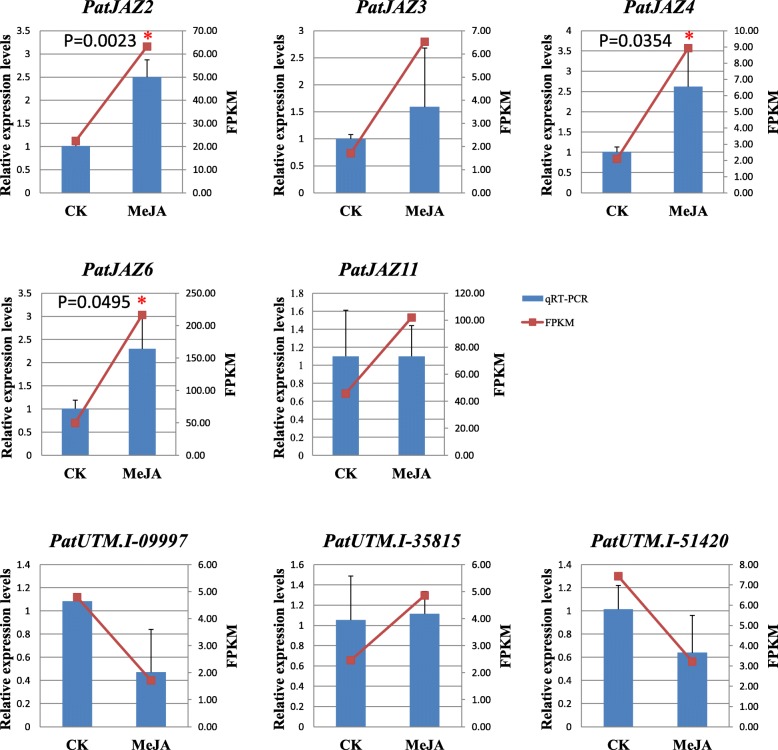
Validation of expression patterns of JAZ proteins and ERFs by qRT-PCR. The relative expression levels were calculated according to the 2^-ΔΔCT^ method using *Pat18S* as internal reference gene. Error bars represent standard deviations. * indicates significant difference of the means at *p* < 0.05 between non-treantment (CK) and MeJA-treatment leaves for each parameter mesureed (*n* = 3). The relative expression of qRT-PCR is indicated on the left y-axis and the normalized expression level (FPKM) of RNA sequencing is indicated on the right y-axis

We also determined the transcription levels of key genes involved in the JA biosynthesis and signaling pathway as well as their expression patterns under exogenous MeJA treatment. Genes having a fold change ratio ≥ 1.5, with corrected *p*-value<0.05 were used for further analysis. JAR1, which converts JA into the biologically active jasmonyl-iso-leucine (JA-Ile), is the key synthases in jasmonate-amino acid synthesis. We observed that patchouli contained 21 JAR1 co-orthologs (Additional file 10: Table S7), and four of them were differently expressed under MeJA treatment (Fig. 5a). PatUTM.I-23903 (8.55) and PatUTM.I-40660 (11.97) were markly down-regulated under MeJA treatment and another two with a more than 1.5-fold up-regulated in expression were identified. CORONATINE INSENSITIVE1 (COI1), an F-box protein acting as a receptor for jasmonate, was demonstrated to directly bind to JA-Ile and coronatine (COR) [29]. We found that eight UniTransModel. Isoforms were annotated as COI1 (Additional file 11: Table S8), however, none were significantly differentially expressed. The SCF^COI1^ complex recognizes JASMONATE ZIM DOMAIN (JAZ) whose degradation enables TFs (such as MYC2) release from complex. There are 14 JAZ UniTransModel. Isoforms were significantly upregulated following application of MeJA (Fig. 5a). In the present study, twelve JAZ genes were recognized (Additional file 12: Figure S4) and cloned, of which five (JAZ2, 3, 4, 6, 11) were further confirmed by qRT-PCR (Fig. 6). Our work indicated that these five JAZs may serve as transcriptional regulatory factors in the JA signaling pathway, which establishes research foundation for further dissection of the molecular mechanism clearly.

TFs of the bHLH (basic/helix-loop-helix) and ERE (ethylene responsive element) families have been previously identified as key regulatory factors in plant JA signaling processes. In this study, we surveyed the bHLHs and AP2/ERFs, and results showed that 67 of them were expressed in 73 bHLH UniTransModel. Isoforms, of which including one bHLH-MYC2 homolog (PatUTM.I-27152, 2.46) was up-regulated and 8 weredown-regulated with a more than 1.5-fold change (Fig. 5b and Additional file 13 Table S9). Six of the down-regulated bHLH were PIF homologs (PatUTM.I-08185, PatUTM.I-09729, PatUTM.I-53598, PatUTM.I-61036, PatUTM.I-07628 and PatUTM.I-08186). On the other hand, we had surveyed the expression patterns of other eleven MYC homologs and found that all of them were up-regulated in response to MeJA treatment (Additional file 14: Figure S5), suggesting that these bHLH transcription factors (MYCs) might be released from JAZ-like repressors, activating transcriptional and expression levels of downstream genes which in charge of the biosynthesis of secondary metabolites. Furthermore, five AP2/ERF TFs were detected as MeJA-responsive genes: PatUTM.I-28888 was significantly upregulated while the rest four were downregulated 1.85 to 4.23 folds. (Fig. 5b and Additional file 13 Table S9). Besides, three AP2/ERF TFs were selected for qRT-PCR analysis: PatUTM.I-09997, PatUTM.I-35815 and PatUTM.I-51420 (Fig. 6). Additionally, MYB and bZIP TFs were also differentially expressed under MeJA treatment (Fig. 5b and Additional file 13 Table S9).

## Discussion

Patchouli was brought into China for its great value of perfume and medicinal industries, and currently is widespread in southern China. According to the morphological characters and proteins patterns, it was classified to three cultivars as follow: *Pogostemon cablin* (Blanco) Benth. cv. shipaiensis, *P. cablin* (Blanco) Benth. cv. gaoyaoensis, and *P. cablin* (Blanco) Benth. cv. zhanjiangensis. *P. cablin* populations have evolved diverse morphological and metabolism characteristics, because of cultivation under varying environmental conditions in different localities in China. Distinct stumpy and rhombic square stem was observed in *P. cablin cv.* ‘*Zhanxiang*’, which also possessed swelling at the nodes and rather small ramification angle compared with another Chinese *P. cablin* landrace cultivated in *yangjiang* [25]. Additionally, in order to have a comprehensive understanding of chemical components in *P. cablin cv.* ‘*Zhanxiang*’, we made a survey with a variety of solvents and detected by GC-MS, results showed that thirty-nine compounds were identified in in *P. cablin*, which mainly were sesquiterpenes. The investigation of morphological and metabolic characteristics provides insights into its biological foundation for resource utilization.

Previous studies on *P. cablin* transcriptome were mainly based on NGS method, but the short reads resulting from that methodology have prohibited the accurate reconstruction of full-length reads without a reference genome sequence [13, 14]. PacBio Iso-seq, the most popular application of third-generation sequencing, has proven useful for accurately characterizing the diverse landscape of isoforms due to its advantage of obtaining full-length transcripts [30]. Utilizing this superior methodology, this study provides the first comprehensive set of full-length isoforms in *P. cablin*. We generated 9.7 G clean data, including 502,101 ROIs and 379,146 FLNC reads. A total of 251,015 consensus transcripts were clustered from the FLNC reads, which included 26,155 HQ isoforms. Except from self-correction, we corrected the consensus transcripts using publicly available Illumina short reads to improve the long read accuracy [31]. After removing redundant sequences, we subjected this polished consensus to a new and innovative pipeline (Cogent: Coding GENome Reconstruction Tool) to merge transcripts to partition gene families and reconstruct 82,335 full-length transcript models.

Based on these highly accurate transcripts, a total of 79.95% UniTransModels were successfully annotated to known proteins in at least one out of the seven functional databases (Nr, SwissProt, KEGG, KOG, GO, Nt and Pfam) and 73.7% of CDSs were annotated with hits in the UniProtKB. KEGG pathway annotation also revealed that 395 transcripts were assigned to the “metabolism of terpenoids and polyketides” in our full-length sequences. Additionally, 15,803 candidate lncRNA and 3176 TFs (belonging to 90 different families) were also obtained. These results in our study also indicated the high capacity of PacBio transcriptome sequencing to generate full-length transcript sequence information, which is of great use and importance for genome annotation and gene function analysis.

Extensive works as well as the chemical components survey in this study have confirmed that *P. cablin* is rich in sesquiterpenes, of which patchoulol is the major component and contributes largely to the clinical effects, and its synthesis pathway has attracted extensive interests. Previous studies using NGS technology have roughly mined the genes related to the synthesis pathway of patchoulol [13], however, due to the technical restriction of NGS, few of them have the full-length sequences, which may lead to the inaccurate gene annotation and restrict the gene function study. Therefore, it still needs a more accurate and comprehensively study and the full-length transcript sequence information in this present work will help a lot. It has become evident that, all terpenoids including patchoulol are derived from IPP and its allylic DMAPP using the MVA and MEP pathways [32]. In this patchouli full-length transcriptome, a total of 102 transcripts encoding enzymes related to patchoulol biosynthesis were identified, including 32 encoding six enzymes in MVA pathway, 39 encoding seven enzymes in MEP pathway, 8 encoding IDI, 4 encoding FDPS and 19 encoding PatPTS, the key enzyme directly responsible for the production of patchouli alcohol in *P. cablin*.

Previous studies have proved that the important plant hormone JA can remarkably improve the biosynthesis of some secondary metabolites in plant; for instance, JA up-regulated TIA biosynthesis in *Catharanthus roseus* [33], artemisinin biosynthesis in *Artemisia annua* [34, 35], and tanshinone biosynthesis in *Salvia miltiorrhiza* [28]. In the present work, after MeJA treatment, we also found the significant increasing of patchoulol content in *P. cablin* leaves. Multiple studies have manifested the enhancement of concentration of terpenoids in response to various abiotic stresses is often mediated by an increase in transcriptional activity of the specific terpenoid biosynthetic genes [36–38]. So we analyzed the transcriptome profiling of patchouli induced by MeJA and found that, most of genes related to the patchoulol biosynthesis were mainly up-regulated expression under MeJA treatment, which accorded with the increasing tendency of patchoulol content. Particularly, DEGs including HMGR_1, HMGR_2, DXS_ (4, 6, 8), HDR_8 and PTS_ (2, 7, 8, 19) genes were up-regulated with at least two-fold change after MeJA treatment. All know that HMGR catalyzes the conversion of HMG-CoA to mevalonate, the rate-limiting step in the MVA pathway. Previous studies have demonstrated the crucial regulatory role of HMGR in sesquiterpenoids biosynthesis, although flux control often involves additional downstream enzymes like sesquiterpene synthases [39, 40]. DXS catalyzes the first reaction in the MEP pathway, it functions as an important regulatory and rate-limiting enzyme in the biosynthesis of plastidial terpenes [32], up- or down-regulation of DXS would increase or decrease the accumulation of specific isoprenoid final products in plants such as arabidopsis [41], tomato [42] and potato [43]. HDR also plays an important role in controlling the production of MEP-derived precursors [44], the alteration of HDR gene expression level might directly influence the synthesis of IPP and DMAPP. Patchoulol synthase (PatPTS) is the key enzyme directly catalyzed the production of patchoulol, many studies have confirmed the relation between PatPTS expression level and the accumulation of patchoulol content in *P. cablin* [45]. In view of the important roles of the above DEGs in controlling carbon flux distribution, it indicated that they mainly participated in MeJA promoting the synthesis of patchoulol. Therefore, it was apparent that MeJA activates the expression of genes related to patchoulol biosynthesis pathway and consequently promoted the production of patchoulol in *P. cablin* leaves; however, the specific regulation mechanisms still need further investigation.

Furthermore, numerous studies have reported on the important roles of TFs in JA signaling pathway, TFs like the bHLH family members MYCs [35, 46] and MYBs [47, 48] as well as EFR family members AP2/ERFs [33, 49] have been identified as key regulators of JA signaling. When jasmonates accumulate and be perceived, the degradation of JAZ proteins liberates the repression of various TFs that execute JA responses. To date, some TFs regulated by the jasmonates hormone signaling cascade that activate the transcription of sesquiterpenoid biosynthesis genes have already been reported [23], however, studies on the TFs participated in JA regulation of PA biosynthesis in patchouli are scarce. In the present study, transcriptome profiling analysis of crucial genes take part in JA biosynthesis and signaling pathways revealed that, two *JAR1* genes and 14 *JAZ* genes were up-regulated under MeJA treatment. Additionally, nine bHLHs were also markedly induced by MeJA, including one MYC which may play important regulatory roles in secondary metabolite biosynthesis. On the other hand, five AP2/ERFs, seven MYBs and 13 bZIPswere found to be MeJA-responsive TFs in *P. cablin*. However, additional researches on the detailed molecular mechanism still need to be carried out.

## Conclusions

The current study comprehensively reported the morphological characterization, volatile chemical compositions and transcriptome characterization of the Chinese-cultivated *P. cablin cv.* ‘*Zhanxiang*’. The established full-length transcriptome provides a fully characterization of patchouli and offer a valuable genetic resource for further study in patchouli. Our MeJA-induced transcriptome profiling analysis also deepens the understanding of the MeJA regulatory mechanism on patchouli alcohol biosynthesis.

## Methods

### Plant material and RNA isolation

The cutting branches of *Pogostemon cablin* (Blanco) Benth*.* cv. *zhangjiangensis* were collected from ‘Shizhen’ Mountain, Guangzhou University of Traditional Chinese Medicine (23.03°N, 113.23°E), which are from twigs of single plant. The rooting and culturing of cutting twigs were performed in a growth chamber in the Research Center of Chinese Herbal Resource Science and Engineering, Guangzhou University of Chinese Medicine. The original plant was identified by Professor Ruoting Zhan of the Research Center of Chinese Herbal Resource Science and Engineering and a voucher specimen was deposited in the Research Center of Chinese Herbal Resource Science and Engineering.

Leaves and stems of *Pogostemon cablin* (Blanco) Benth*.* cv. *zhanjiangensis* were harvested, then snap-frozen via liquid nitrogen after a quick rinse by distilled water.

For hormone treatment, 300 μM MeJA (Sigma-Aldrich) was used to spray on the leaves, whereas 0.5% ethanol was used as a negative control. Leaf samples were washed and collected at 8 h after spraying with three biological replicates. All plants used in this study were at the 4-leaf stage of growth.

The GeneMark Plant Total RNA Purification Kit (GeneMarkBio, Taiwan) was used to extract RNA from patchouli tissues, and then an Agilent 2100 Bioanalyzer (Agilent Technologies, Santa Clara, CA) was used to measure the RNA integrity and a Qubit Fluorometer (Thermo Fisher Scientific) was used to estimate the RNA concentration. High-quality mRNA isolation was performed via the oligo d(T) magnetic beads and further processed for cDNA preparation.

### cDNA construction and PacBio Iso-Seq

According to the PacBio Isoform Sequencing protocol, the Clontech SMARTer PCR cDNA Synthesis kit (Clontech, Mountain View, CA, USA) was used for cDNA synthesis and library construction. The full-length cDNA Iso-Seq template was sequenced in two SMRT cells on a PacBio Sequel System after purification, size selection, re-amplification and SMRTbell template preparation. Size selection was performed via a BluePippin (Sage Science, Beverly, MA) to construct a cDNA library of size > 4 kb. The same amount of non-selected cDNA and > 4 kb cDNA were combined to form Iso-Seq library for SMRT sequencing.

### Iso-Seq data processing with standard bioinformatics pipeline

Raw sequencing data were processed using the SMRTlink4.0 software through standard Iso-Seq protocol. First, reads of insert (ROIs) were generated from subreads removing adapters and artifacts, and then classified into full-length nonchimeric (FLNC) and non-full-length (nFL) reads by detecting primer and polyA tail with ‘pbclassify.py’. Clustering of the full-length transcripts was performed via Iterative Clustering for Error Correction (ICE) algorithm. Followed by final Arrow polishing, the Quiver algorithm was used to polish and categorize the consensus sequences produced above using nFL reals. Error correction was performed via ‘proovread (2.13.12)’ [31, 50] using a Illumina RNA-seq dataset.

### Full-length unique transcript model reconstruction

Redundancy isforms were removed using the program CD-HIT (v4.6.8) [26] to generate a high-quality transcripts dataset for patchouli by applying the all error-corrected full-length polished consensus transcripts. In Cogent (Coding GENome Reconstruction Tool), above non-redundant transcripts were submitted to create its k-mer profile and calculate pairwise distance, and clustered into families with their k-mer similarity. One or several unique transcript model(s) (UniTransModels) were reconstructed from each of the resultant transcript families via a De Bruijn graph method [22].

### Functional annotation of UniTransModels

The obtained UniTransModels were mapped to seven databases to acquire the annotation information. We used the software BLAST and set the e-value ‘1e-5’ in Nt (Nucleotide database) database analysis and used software Hmmscan in Pfam (Protein family) database analysis. Nr (Non-Redundant Protein Database), KEGG (Kyoto Encyclopedia of Genes and Genomes), KOG (Cluster of Orthologous Group), Swiss-Prot and and GO (Gene Ontology) databases annotations were conducted using BLASTX with a cut-off e-value of ‘1e-5’.

### Prediction and further annotation of coding sequences

To determine protein coding sequences (CDS) from cDNAs, UniTransModels were processed via ANGEL pipeline using this species or closely related species’ confident protein sequences for ANGEL training and prediction [51]. The predicted CDS were then used to search and confirm against three well-characterized protein databases: UniProtKB (UniProt Knowledgebase)_Viridiplantae, UniProtKB_*Arabidopsis thaliana* and UniProtKB_Rice (including all green plant, Arabidopsis and rice curated protein entries, respectively) via software BLASTX and set the e-value ‘1e-5′.

### Identification of transcription factors and LncRNA

The iTAK software was used to identify and classify TFs (transcription factors) and TRs (transcription regulators) in *P. cablin* [52]. The identified transcripts of each gene family were compiled and the number was compared with those in *A. thaliana* and *O. stativa* (the data were retrieved from iTAK: Plant Transcription factor & Protein Kinase Identifier and Classifier, http://itak.feilab.net/cgi-bin/itak/index.cgi).

In this study, the transcripts were applied to the Pfam, CNCI (Coding-Non-Coding Index) and CPC (Coding Potential Calculator) databases for lncRNA prediction through screening coding potential. To predict potential miRNA precursors (pre-miRNAs), the searchable miRNA database miRBase (www.mirbase.org) was used to select hits from the candidate lncRNAs which were similar (sequence coverage > 90%) with published miRNA sequences [53, 54].

### Illumina library construction and sequencing

Leaves treated with MeJA were harvested and used for total RNAs extraction. RNAs were sequenced with three biological replicates via an Illumina HiSeq 2500 platform (Illumina Inc., CA, USA), and the obtained sequences were mapped to the patchouli reference full-length UniTransModels built in this study. The amount of fragments mapping to each transcript was counted and used to determine each transcript’s relative expression level, and FPKMs (fragments per kilobase of transcript per million mapped reads) method was used to normalize the relative gene expression levels. Genes having a fold change ratio ≥ 2, with corrected *p*-value<0.01 were defined as differentially expressed genes (DEGs).

### Determination of the volatile chemical composition using gas chromatography mass spectrometry (GC-MS)

Leaf samples of *P. cablin* were extracted using four different solvents, i.e. chloroform, ethyl alcohol, ethyl acetate, and n-Hexane. A HP6890/5973 GC–MS (Agilent Technologies, Palo Alto, CA, USA) was used to analyze the extracted with a split ratio of 10:1, an injection temperature of 230 °C, an interface temperature of 280 °C and ion source temperature was 230 °C. Separation of compounds was performed on DB-FFAP capillary column (30 m × 0.25 mm × 0.25 μm). The flow rate of Helium carrier gas was set at 1.0 mL∙min^− 1^. The oven temperature was set to 50 °C initially, then heating of 20 °C∙min^− 1^ to 130 °C and a final 1 min at 130 °C. Follow by ramping to 150 °C at 2 °C∙min^− 1^ and held for 5 min, and ramping at 20 °C∙min^− 1^ to 230 °C. Electron impact ionization mode of the mass spectrometric was applied to record the mass spectra at an ionizing energy of 70 eV in scanning rang of *m/z* 29–500. The spectra peaks were identified via WILEY7n.L (Palisade Corporation, N.Y., USA) and Nist08.L (NIST, Gaithersburg, Md., USA) databases, and data normalization were performed with fresh weight of each sample and external standard.

### Patchoulol exaction and analysis

Approximately, 0.25 g of leaf samples extracted with 50 mL Ethyl acetate using an ultrasonic cleaner for 20 min, repeat two times, and then concentrated by rotary evaporation. Concentrate dissolved in hexane to a volume of 5 mL. The extraction was analyzed using Agilent 7890B Gas Chromatograph with 5977A inert Mass Selective Detector (Agilent, United States). The injection volume is 1 μl of continuous filtrate, and helium is carrier gas. Agilent HP-5MS column (30 m × 250 mm × 0.25 mm film thickness) as a column for separation. GC oven temperature was programmed at an initial temperature of 50 °C for 2 min with an increase of 20 °C/min to 130 °C, and raised to 150 °C at a rate of 2 °C / min for 5 min. And temperature was then raised to 230 °C at a rate of 20 °C / min. Quadrupole and ion source temperatures are set to 150 °C and 230 °C. NIST14/Wiley275Mass Spectral Library was used for metabolite identification. This study used the external standard method to quantify the patchouli alcohol; there were three biological replicates and two technical replicates for each tissue.

### Quantitative real-time PCR

RNA extraction was performed as described above, and first-strand cDNA synthesis was used HiScript II Q RT SuperMix for qPCR (+gDNA wiper) (Vazyme Biotech Co., Ltd.). The primers in these experiments were designed via Primer Premier 5.0 and were listed in Additional file 15: Table S10. QRT-PCRs for genes were carried out with three biological replicates and two technical replicates by ChamQ Universal SYBR qPCR Master Mix (Vazyme Biotech) on CFX96 Real-Time PCR Detection System (Bio-Rad, United States). Then, the reference gene Pat18s was served as control for normalization and calculated the transcript levels of target genes via the 2^-△△Ct^ method.

## Additional files

Additional file 1: **Figure S1.** Morphology characteristics of different *Pogostemon cablin* cultivars, including **(a)** *P. cablin cv*. ‘*Zhanxiang*’, **(b)** *P. cablin cv*. ‘*Yangjiang*’, and **(c)** *P. cablin cv*. ‘*Indonesia*’. (DOCX 458 kb)

Additional file 2: **Figure S2.** Analyses of the volatile chemical components of *P. cablin* using gas chromatography mass spectrometry. **a** Eluted time and Mass-to-charge (m/z) of patchouli alcohol. **b** Eluted time and Mass-to-charge(m/z) of pogostone. **c** GC-MS base peak chloroform extract of *P. cablin. (DOCX 433 kb)*

Additional file 3: **Table S1.** Comparison results between SMRT sequencing and Illumina sequencing. (XLSX 9 kb)

Additional file 4: **Figure S3.** Homologous species distribution of *P. cablin* annotated in Nr database. (DOCX 152 kb)

Additional file 5: **Table S2.** Annotation of CDS with different protein databases. (XLSX 2631 kb)

Additional file 6: **Table S3.** Known RNA motifs annotated in *P. cablin* lncRNAs. (XLSX 8 kb)

Additional file 7: **Table S4.** Summary of re-characterisation and transcription isoforms of genes annotated in pathways related to terpenoids biosynthesis. (XLSX 22 kb)

Additional file 8: **Table S5.** List of DEGs in *Pogostemon cablin* under MeJA treatment. (XLSX 190 kb)

Additional file 9: **Table S6.** MeJA-induced expression pattern of genes involved in patchoulol biosynthesis. (XLSX 33 kb)

Additional file 10: **Table S7.** Annotation of JAR1 co-orthologs in *Pogostemon cablin*. (XLSX 9 kb)

Additional file 11: **Table S8.** Eight UniTransModel. Isoforms annotated as COI1. (XLSX 9 kb)

Additional file 12: **Figure S4.** Expression changes of JAZ proteins in *Pogostemon cablin* induced by MeJA. The heat map showing log_2_(FPKM+ 1) values of each protein. (DOCX 126 kb)

Additional file 13: **Table S9.** Expression profiles of MeJA-responsive genes associated with JA synthesis and signaling in patchouli. (XLSX 24 kb)

Additional file 14: **Figure S5.** Expression changes of *PatMYCs* in *Pogostemon cablin* induced by MeJA. (DOCX 184 kb)

Additional file 15: **Table S10.** Primers used for qPCR analysis. (XLSX 9 kb)

## Data Availability

The datasets supporting the conclusions of this article are included within the article and its Additional files. The PcaBio SMRT reads and the Illumina RNA-seq data generated in this study have been submitted to the NCBI Sequence Read Archive (SRA; http://www.ncbi.nlm.nih.gov/sra). Accession numbers for two SMRT cells of sequencing are SRR8761881 and SRR8761882 under BioProject PRJNA528262. Accession number for Illumina cDNA libraries obtains from the control and samples of leaves treated with MeJA are SRR8756845, SRR8756846, SRR8756847, SRR8755475, SRR8755476, and SRR8755477 under BioProject PRJNA511937.

## Abbreviations

AACT: Acetyl-CoA acyltransferase
AP2/ERF: APETALA2/ethylene response factor
bHLH: basic helix-loop-helix
CDS: Coding sequences
CK: Control
CMK: 4-diphosphocytidyl-2-C-methyl-D-erythritol kinase
Cogent: Coding GENome Reconstruction Tool
COR: Coronatine
DEGs: Differentially expressed genes
DMAPP: Dimethylallyl pyrophosphate
DXR: 1-deoxy-D-xylulose-5-phosphate reductoisomerase
DXS: 1-deoxy-D-xylulose-5-phosphate synthase
ERF: Ethylene responsive factor
FDPS: Farnesyl diphosphate synthase
FDR: False discovery rate.
FLNC: Full-length nonchimeric
FPKMs: Fragment per kilobase of exon model per million mapped reads
FPP: Farnesyl diphosphate
HDR: 1-hydroxy-2-methyl-2-butenyl 4-diphosphate reductase
HDS: 1-hydroxy-2-methyl-2-butenyl 4-diphosphate synthase
HMGR: 3-hydroxy-3-methylglutaryl-CoA reductase
HMGS: 3-hydroxy-3-methylglutaryl-CoA synthase
ICE: Iterative Clustering for Error Correction
IDI: Isopentenyl diphosphate isomerase
JA-Ile: Jasmonyl-iso-leucine
JAZ: Jasmonate ZIM domain-containing protein
lncRNA: Long non-coding RNA
MCT: MEP cytidyltransferase
MDS: 2-C-Methy-D-erythritol 2,4-cyclodiphosphate synthase
MeJA: Methyl jasmonate
MVD: Mevalonate 5-dinhophate decarboxylase
MVK: Mevalonate kinase
MYC: Myelocytomatosis oncogene
nFL: non-full-length
PatPTS: Patchoulol synthase
PMK: Phosphomevalonate kinase
ROIs: Reads of insert
TF: Transcription factor
TR: Transcription regulator
UniTransModels: Unique transcript model(s)
