# Supplementary material for: Full-length transcriptome sequencing and methyl jasmonate-induced expression profile analysis of genes related to patchoulol biosynthesis and regulation in Pogostemon cablin
Source: BMC Plant Biol. 2019 Jun 20;19:266. doi: 10.1186/s12870-019-1884-x (PMC6585090; doi:10.1186/s12870-019-1884-x)
Supplement: Supplementary file 1 — Figure S1. Morphology characteristics of different Pogostemon cablin cultivars, including (a) P. cablin cv. ‘Zhanxiang’, (b) P. cablin cv. ‘Yangjiang’, and (c) P. cablin cv. ‘Indonesia’. (DOCX 458 kb) [file 12870_2019_1884_MOESM1_ESM.docx]

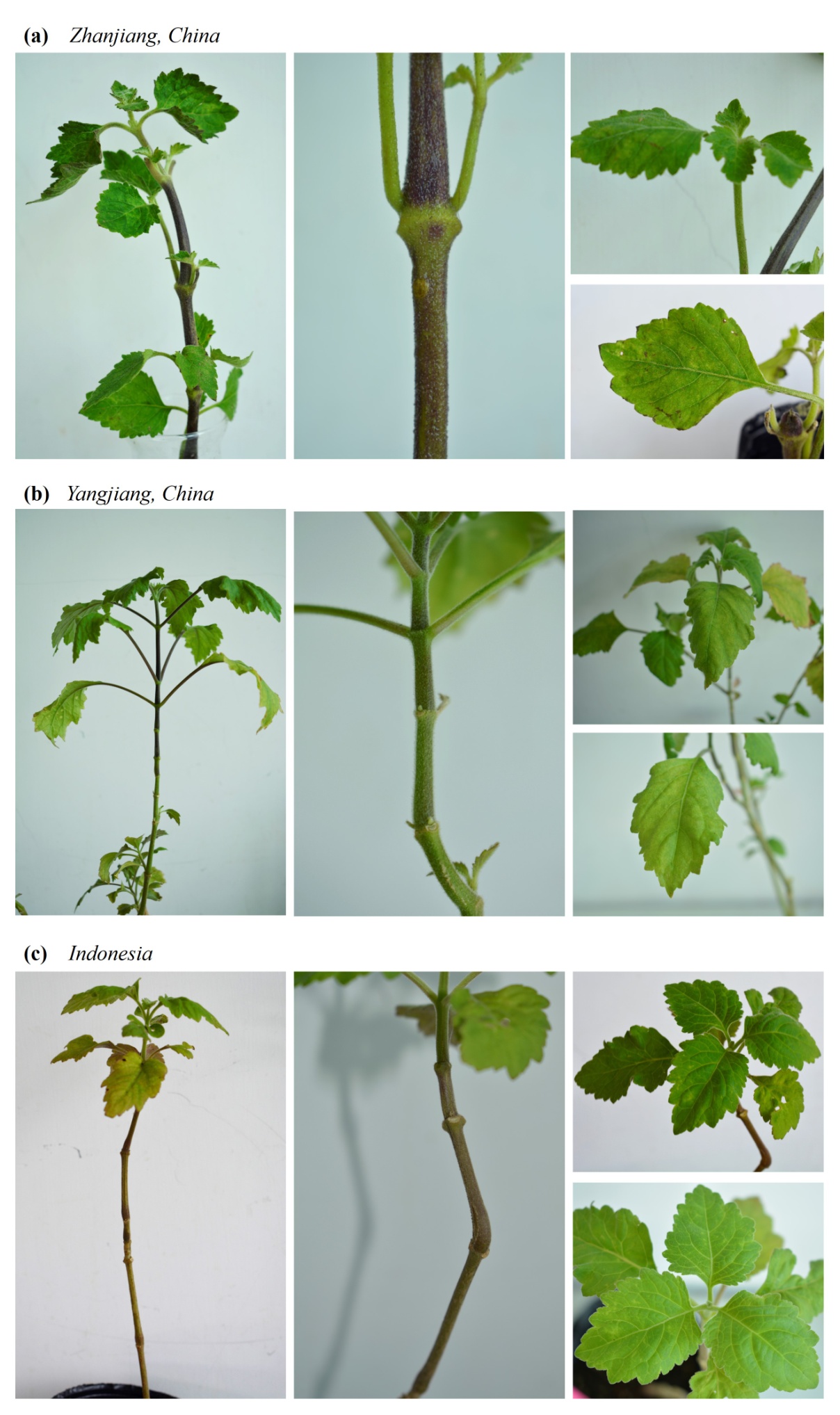


**Fig. S1**  Morphology characteristics of different *Pogostemon cablin* cultivars, including **(a)** *P. cablin cv*. ‘*Zhanxiang*’, **(b)** *P. cablin cv*. ‘*Yangjiang*’, and **(c)** *P. cablin cv*. ‘*Indonesia*’.
