# Supplementary material for: Full-length transcriptome sequencing and methyl jasmonate-induced expression profile analysis of genes related to patchoulol biosynthesis and regulation in Pogostemon cablin
Source: BMC Plant Biol. 2019 Jun 20;19:266. doi: 10.1186/s12870-019-1884-x (PMC6585090; doi:10.1186/s12870-019-1884-x)
Supplement: Supplementary file 2 — Figure S2. Analyses of the volatile chemical components of P. cablin using gas chromatography mass spectrometry. a Eluted time and Mass-to-charge (m/z) of patchouli alcohol. b Eluted time and Mass-to-charge(m/z) of pogostone. c GC-MS base peak chloroform extract of P. cablin. (DOCX 433 kb) [file 12870_2019_1884_MOESM2_ESM.docx]

**(a)**

**(b)**

**(c)**

**Fig. S2** Analyses of the volatile chemical components of *P. cablin* using gas chromatography mass spectrometry. **(a)** Eluted time and Mass-to-charge(m/z) of patchouli alcohol. **(b)** Eluted time and Mass-to-charge(m/z) of pogostone. **(c)** GC-MS base peak chloroform extract of *Pogostemon cablin.*
