# Supplementary figures and images for: Full-length transcriptome sequencing and methyl jasmonate-induced expression profile analysis of genes related to patchoulol biosynthesis and regulation in Pogostemon cablin
Source: BMC Plant Biol. 2019 Jun 20;19:266. doi: 10.1186/s12870-019-1884-x (PMC6585090; doi:10.1186/s12870-019-1884-x)

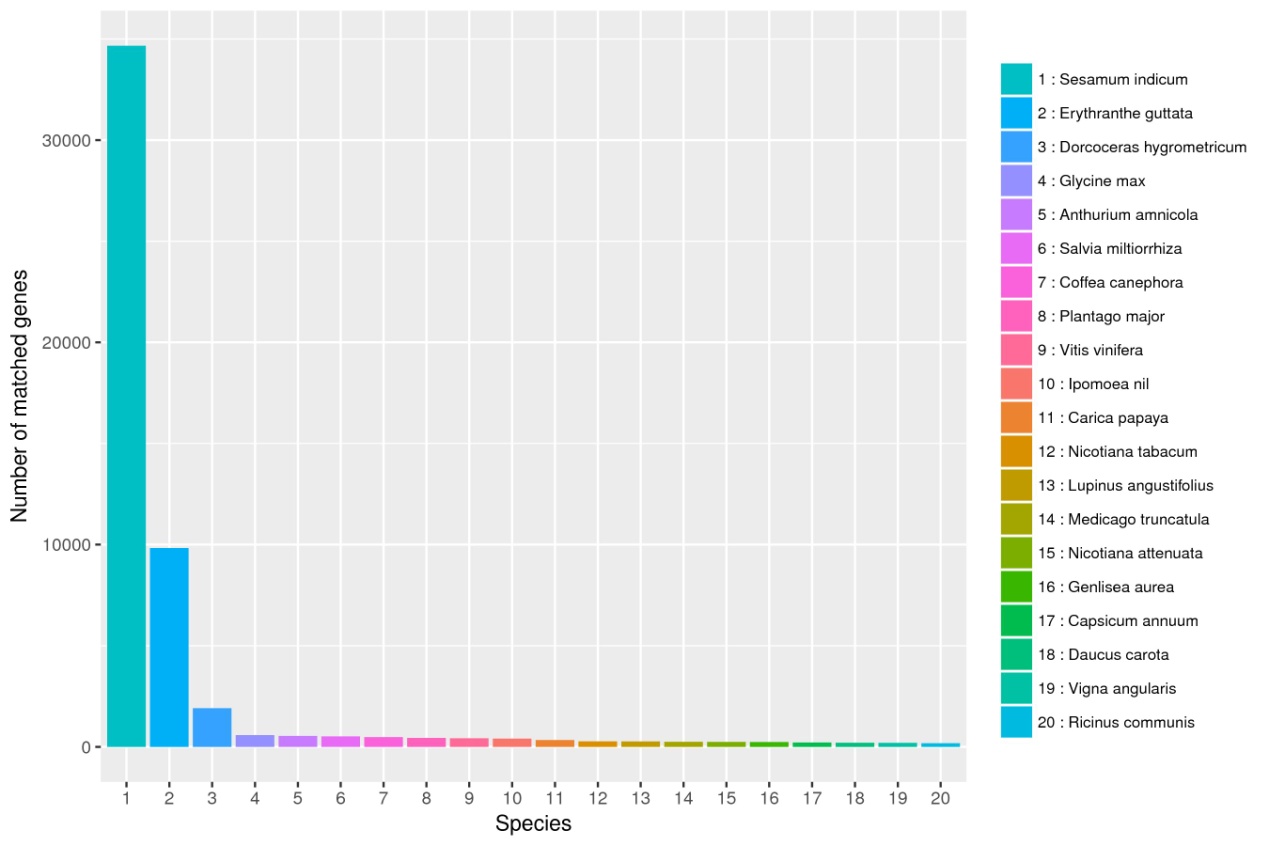


**Fig. S3** Homologous species distribution of *P. cablin* annotated in Nr database.

Supplement: Supplementary file 4 — Figure S3. Homologous species distribution of P. cablin annotated in Nr database. (DOCX 152 kb) [file 12870_2019_1884_MOESM4_ESM.docx]

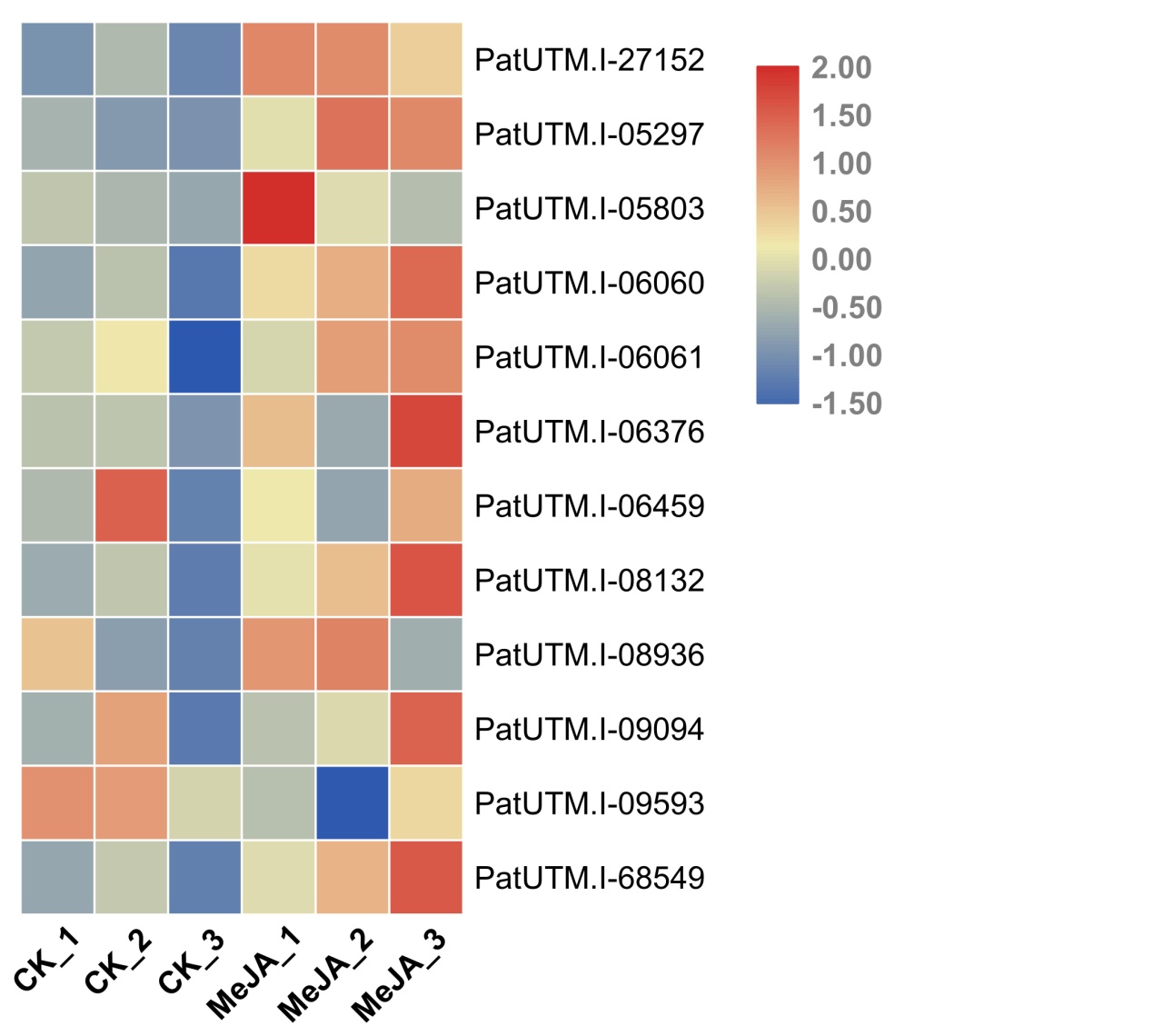


**Fig.S5** Expression changes of *PatMYCs* in *Pogostemon cablin* induced by MeJA.

Supplement: Supplementary file 14 — Figure S5. Expression changes of PatMYCs in Pogostemon cablin induced by MeJA. (DOCX 184 kb) [file 12870_2019_1884_MOESM14_ESM.docx]
