# Supplementary material for: Full-length transcriptome sequencing and methyl jasmonate-induced expression profile analysis of genes related to patchoulol biosynthesis and regulation in Pogostemon cablin
Source: BMC Plant Biol. 2019 Jun 20;19:266. doi: 10.1186/s12870-019-1884-x (PMC6585090; doi:10.1186/s12870-019-1884-x)
Supplement: Supplementary file 12 — Figure S4. Expression changes of JAZ proteins in Pogostemon cablin induced by MeJA. The heat map showing log2(FPKM+ 1) values of each protein. (DOCX 126 kb) [file 12870_2019_1884_MOESM12_ESM.docx]

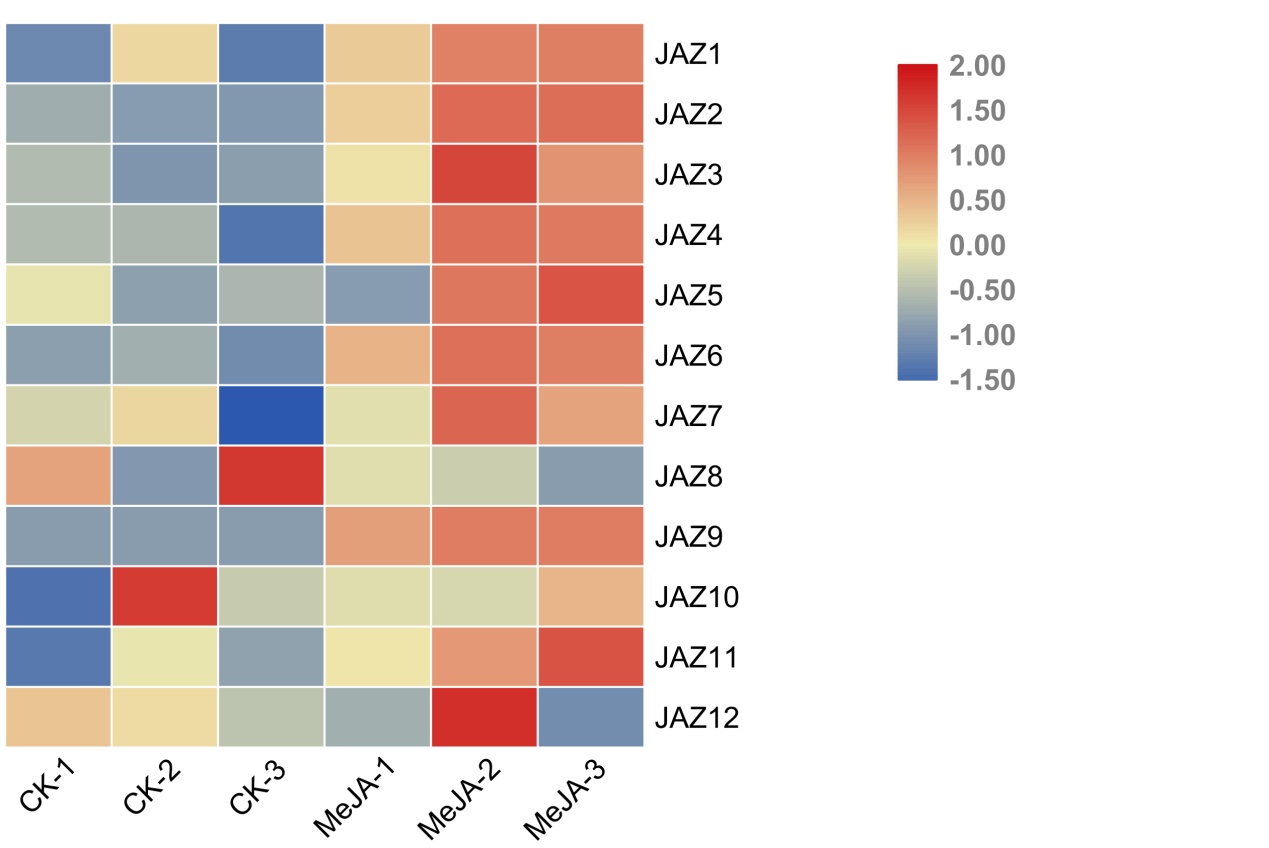


**Fig.S4** Expression changes of JAZ proteins in *P.cablin* induced by MeJA. The heat map showing log_2_(FPKM+1) values of each protein.
